# Detection of Hepatocellular Carcinoma in Contrast-Enhanced Magnetic Resonance Imaging Using Deep Learning Classifier: A Multi-Center Retrospective Study

**DOI:** 10.1038/s41598-020-65875-4

**Published:** 2020-06-11

**Authors:** Junmo Kim, Ji Hye Min, Seon Kyoung Kim, Soo-Yong Shin, Min Woo Lee

**Affiliations:** 1 Department of Health and Sciences and Technology, SAIHST, Sungkyunkwan University, 81, Irwon-ro, Gangnam-gu, Seoul, 06351 Korea; 2 Department of Radiology and Center for Imaging Science, Samsung Medical Center, Sungkyunkwan University School of Medicine, 81, Irwon-ro, Gangnam-gu, Seoul, 06351 Korea; 3 Department of Digital Health, SAIHST, Sungkyunkwan University, 115, Irwon-ro, Gangnam-gu, Seoul, 06351 Korea; 4 Big Data Research Center, Samsung Medical Center, 81, Irwon-ro, Gangnam-gu, Seoul, 06351 Korea

**Keywords:** Cancer screening, Software

## Abstract

Hepatocellular carcinoma (HCC) is one of the most common malignant tumors and a leading cause of cancer-related death worldwide. We propose a fully automated deep learning model to detect HCC using hepatobiliary phase magnetic resonance images from 549 patients who underwent surgical resection. Our model used a fine-tuned convolutional neural network and achieved 87% sensitivity and 93% specificity for the detection of HCCs with an external validation data set (54 patients). We also confirmed whether the lesion detected by our deep learning model is a true lesion using a class activation map.

## Introduction

Primary liver cancer is the fifth most common malignant tumor worldwide and the third most common cause of cancer-related mortality, with hepatocellular carcinoma (HCC) accounting for 85–90% of primary liver cancers^1,2^. Many practice guidelines for HCC management state that tumor size is one of the important prognostic factors in patients with HCC, along with liver function and patient performance status^3,4^. Therefore, earlier detection and diagnosis of HCC would be of paramount importance for better survival outcomes after treatment.

In terms of the diagnosis of HCC, magnetic resonance imaging (MRI) provides higher sensitivity than computed tomography (CT). Currently, gadoxetic acid-enhanced liver MRI is widely used for HCC diagnosis and has shown significantly higher sensitivity than MRI performed with other contrast agents^5^. This improved sensitivity of gadoxetic acid-enhanced MRI is mainly attributed to hepatobiliary phase images as most HCCs (80–90%) are hypointense in this phase^6,7^. However, the per lesion sensitivity for HCC on gadoxetic acid-enhanced MRI was 87% (95% confidence interval: 83–92%) in a recent meta-analysis^5^. This implies that computational decision-support tools may play an important role in improving the diagnostic performance of gadoxetic acid-enhanced MRI^8^.

Deep learning has shown remarkable results in the field of computer vision^9^. Deep learning-based methods have also demonstrated that they are well suited for recognition and classification of medical images^10^ and they can be used as an effective screening tool in medical image analysis^11^. Therefore, deep learning systems can be an auxiliary diagnostic system for the diagnosis of HCC, as well. To our knowledge, however, there are no deep learning-based HCC detection systems using liver MRI in the English literature. Therefore, the purpose of this study was to develop a fully automated deep learning model to detect HCC using hepatobiliary phase MR images in patients who underwent surgical resection for HCC and evaluate its performance in detecting HCC on liver MRI compared to human readers.

## Results

### Our CNN architecture

Tables 1–3 show the experimental results of combinations of heuristically chosen hyperparameters to optimize the CNN architecture for HCC detection in liver MRI. Table 1 shows the results of the comparison of batch normalization (BN)^12^ and dropout^13^ to prevent overfitting. Since training was terminated when there was no improvement in the accuracy of validation datasets within 10 epochs, the number of epochs in each case were different. As a result, BN only showed the best performance.

**Table 1:** Comparison results of combination of batch normalization (BN) and dropout rate. BN only showed the best accuracy.

Regularization Layer	Kernel size	Activation function	Optimizer	Number of Epochs	Accuracy
BN	2 × 2	ReLU^a^	Adam^b^	46	93.7%
BN and Dropout (0.1)^c^	2 × 2	ReLU	Adam	28	74.6%
BN and Dropout (0.2)	2 × 2	ReLU	Adam	165	92.7%
BN and Dropout (0.3)	2 × 2	ReLU	Adam	48	86.6%
BN and Dropout (0.4)	2 × 2	ReLU	Adam	46	85.0%
BN and Dropout (0.5)	2 × 2	ReLU	Adam	55	73.9%
Dropout (0.1)	2 × 2	ReLU	Adam	59	86.6%
Dropout (0.2)	2 × 2	ReLU	Adam	116	88.2%
Dropout (0.3)	2 × 2	ReLU	Adam	96	87.7%
Dropout (0.4)	2 × 2	ReLU	Adam	112	87.9%
Dropout (0.5)	2 × 2	ReLU	Adam	28	72.1%

^a^ReLU: rectified linear unit.

^b^Adam: Adam optimizer.

^c^Dropout (0.1) indicates a dropout rate of 0.1.

**Table 2:** Comparison of diverse activation functions. ReLU showed the best accuracy.

Activation function	Number of Epochs	Accuracy
LeakyReLU (0.1)^a^	37	84.2%
PReLU^b^	28	90.4%
ELU (0.1)^c^	73	91.6%
ReLU^d^	46	93.7%

^a^LeakyReLU: leaky rectified linear function.

^b^PReLU: parametric ReLU.

^c^ELU: exponential linear unit.

^d^ReLU: rectified linear unit.

**Table 3:** Comparison of diverse optimizers. Optimization with Adam had the best performance.

Optimizer	Learning Rate	Number of Epochs	Validation loss	Accuracy
AdaGrad^a^	0.001	23	0.83	55.7%
Adam^b^	0.001	46	0.18	93.7%
RMSprop^c^	0.001	62	0.18	91.4%
SGD^d^	0.001	14	0.98	58.7%

^a^AdaGrad: adaptive gradient algorithm.

^b^Adam: a method for stochastic optimization.

^c^RMSprop: a mini-batch version of rprop.

^d^SGD: stochastic gradient descent.

To solve the vanishing gradient problem, various activation functions, including the leaky rectified linear function (LeakyReLU)^14^, a Parametric Rectified Linear Unit (PReLU)^15^, Exponential Linear Units (ELUs)^16^, and Rectified Linear Unit (ReLU)^17^ were compared. Among these activation functions, ReLU showed the best performance (Table 2).

**Figure 1:**
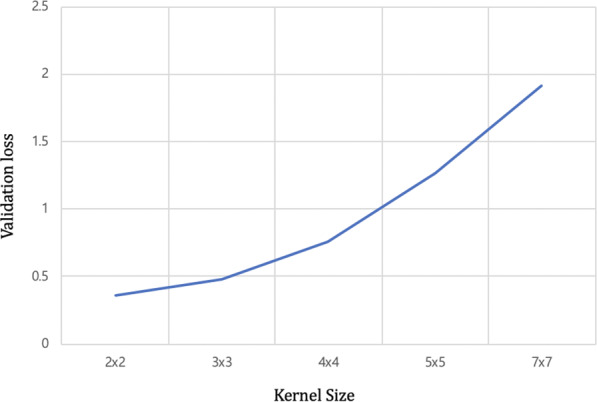
Comparison results of diverse kernel size. Kernel size with 2 × 2 showed the smallest loss.

To minimize information loss, we prefixed the stride as 1 and then changed the kernel size from 2 × 2 to 7 × 7. As shown in Fig. 1., the 2 × 2 kernel achieved the minimum validation loss. To choose the right optimizer for the lowest possible error and steady learning speed, we compared optimization functions, including the adaptive gradient algorithm (AdaGrad)^18^, a method for stochastic optimization (Adam)^19^, a mini-batch version of rprop (RMSprop)^20^, and stochastic gradient descent (SGD)^21^. The Adam optimizer was found to be the most accurate optimization function that affected learning speed and probability (Table 3).

**Figure 2:**
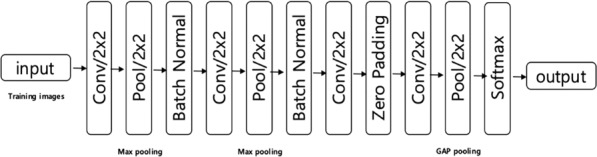
CNN structure of our model for HCC detection in MR images.

The CNN components included widely used components such as the convolution filter, pooling, BN, dropout, padding, activation functions, and optimization functions. Each component also had diverse parameters, such as convolution filter size, pooling method, and stride size. Figure 2 shows the designed CNN architecture.

### Performance

**Figure 3:**
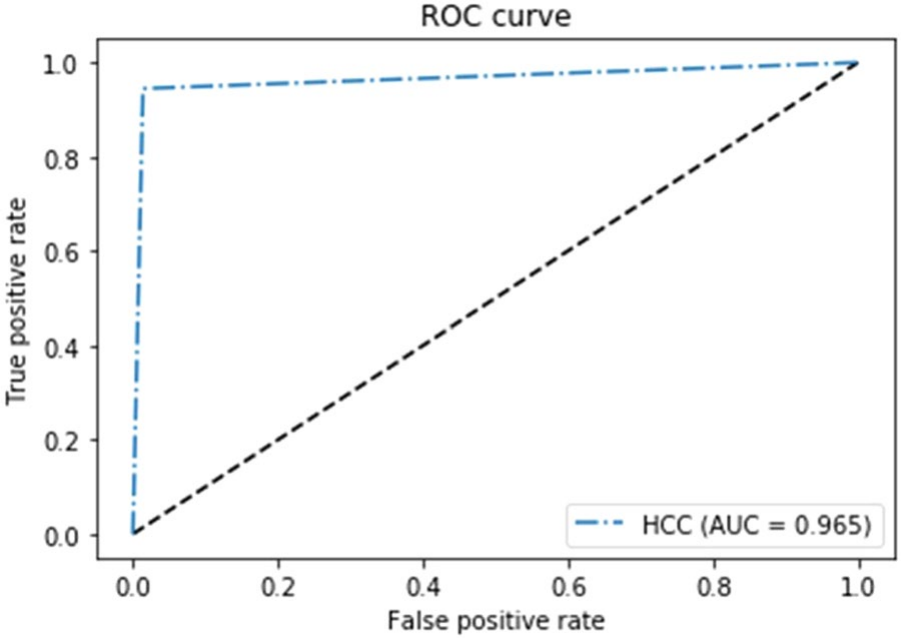
ROC analysis for the proposed model in test datasets.

**Figure 4:**
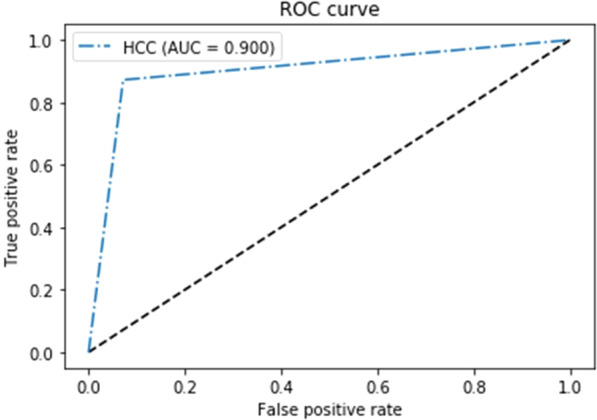
ROC analysis for the proposed model in external validation datasets.

**Figure 5:**
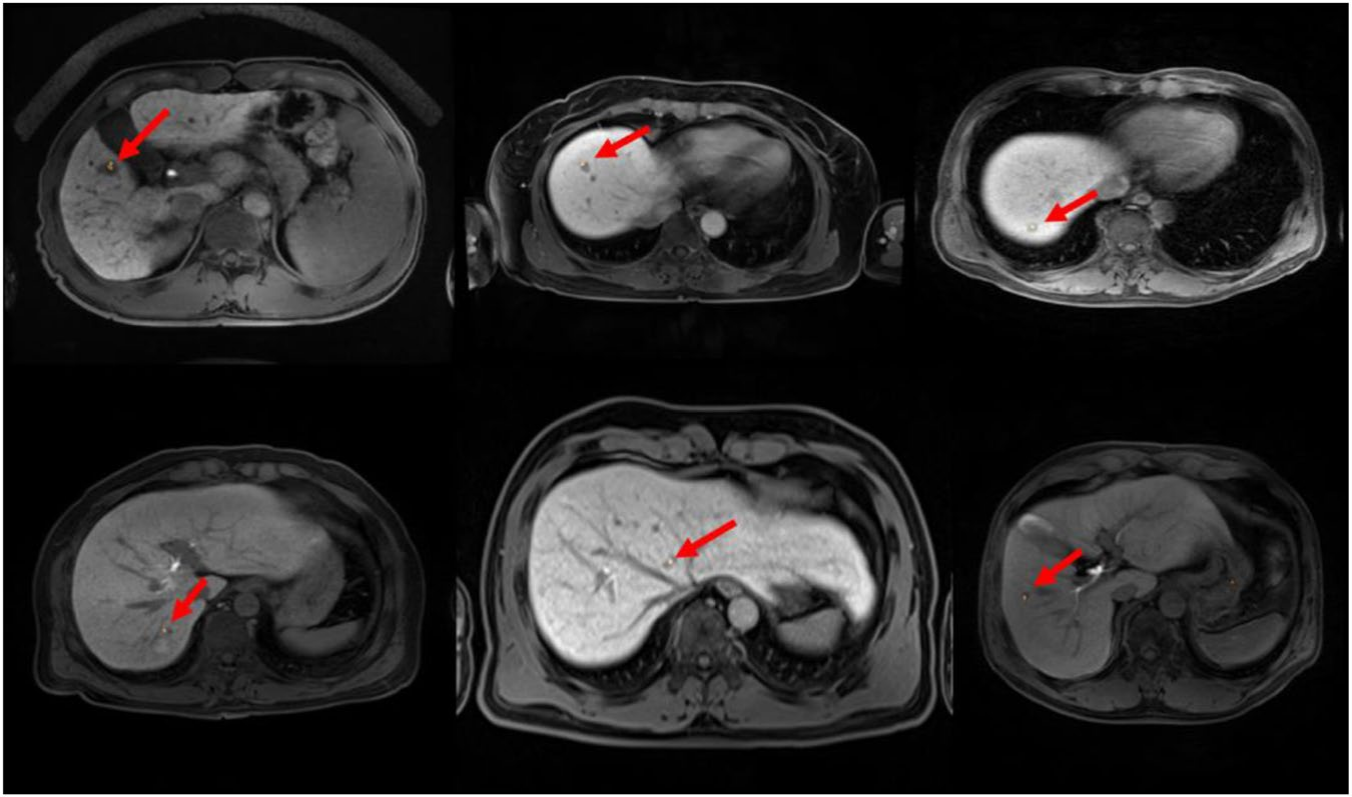
True positive detection (arrow) of HCC by our trained model showing the area of interest of the trained model using CAM method. Although the expert radiologist was able to detect these small HCCs, the less experienced radiologist did not find them. Also, it took longer time for both human readers to detect these small HCCs compared to our model.

**Figure 6:**
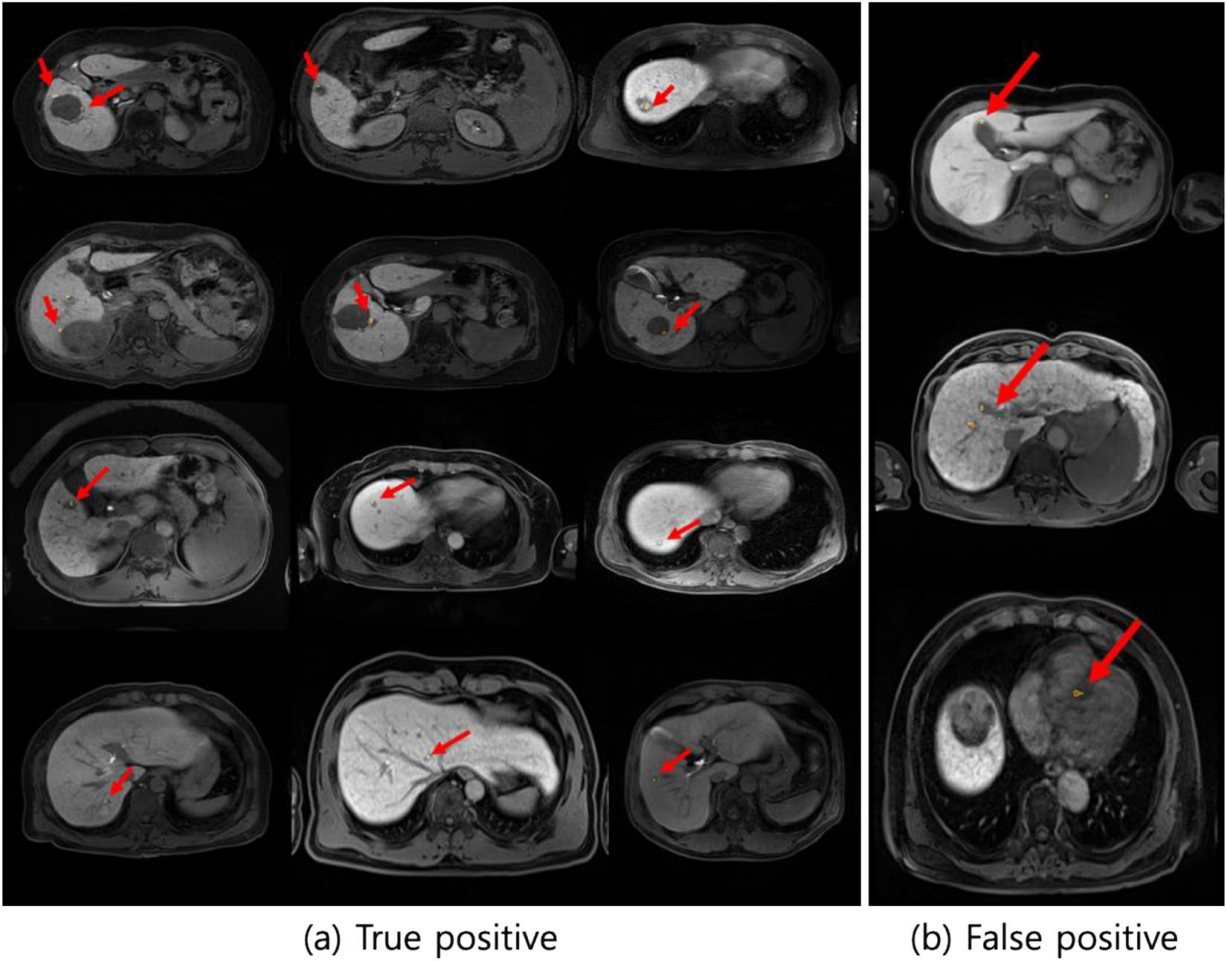
Examples of true positive and false positive detections (arrow) of HCC by our trained model using CAM method. A physician can intuitively discriminate true HCC from pseudo lesions based on HCC candidate indicated by color map.

The optimized CNN architecture achieved 94% sensitivity, 99% specificity, and 0.97 area under curve (AUC) for HCC cases in the test dataset (Fig. 3) and achieved 87% sensitivity and 93% specificity and an AUC of 0.90 for external validation datasets (Fig. 4). The mean size of HCCs that were missed by the less experienced radiologist but detected by our model was 1 ± 0.2 cm (Fig. 5). Therefore, our model seemed to have advantages over the performance of the less experienced radiologist in detecting very small HCCs (Fig. 5). However, our model showed false positive detections including the gallbladder, blood vessels, and heart (Fig. 6). The overall performance was not significantly different between our model and the less experienced radiologist (Table 4).

**Table 4:** Comparison between proposed model and human performance in external validation dataset.

Class	Sensitivity	Specificity	Accuracy	Interpretation time per 100 images (seconds)
Model	0.87	0.93	0.90	3.4
Human^a^	0.86	0.92	0.91	18
Human^b^	0.98	0.93	0.94	18

^a^less experienced radiologist.

^b^expert radiologist.

In terms of image classification time, our model was much faster than human readers, regardless of the degree of experience (Table 4). Our model took 0.03 seconds to classify one image and the average image classification time of 100 images in a single patient was 3.4 seconds using a commercial PC (3.8 GHz Intel Core i5, 16 GB RAM, Radeon Pro 580 8 G). We used the CPU version considering the PC without an expensive GPU usually used in the doctor’s office. In comparison, it took 0.18 seconds to classify one image and average image classification time of 100 images in a single patient was 18 seconds in both radiologists.

## Discussion

**Figure 7:**
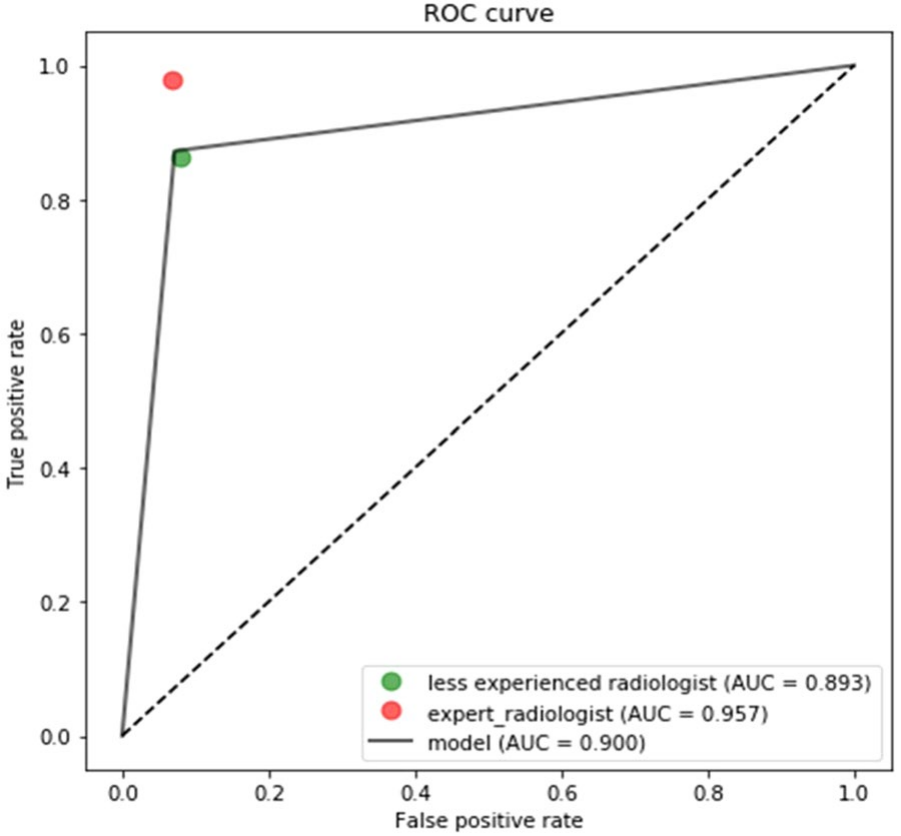
ROC analysis for the proposed model and human readers in external validation dataset.

Recently, deep learning has gained attention in the field of medical imaging, including radiologic imaging^8,22–24^. In this study, we performed deep learning of the hepatobiliary phase images of 92,645 gadoxetic acid-enhanced MR images using a fine-tuned CNN. External validation using the training generation model for 4,537 images obtained by various MR scanners from multiple vendors showed an 87% sensitivity for HCC, 93% specificity, and an AUC of 0.90. Our model seems to be more sensitive than less experienced radiologists in detecting very small HCCs. Furthermore, the classification time of the HCC nodule was 30 milliseconds per image, approximately six times faster than human readers (180 milliseconds). The accuracy of HCC detection was as high as 90%. Based on these results, our deep learning system may be used as an effective decision-support tool for the detection of small HCCs (i.e., sub centimeter HCCs) particularly by less experienced radiologists (Fig. 7).

However, our model exhibited a not infrequent rate of false positive detection. Contrary to our expectation, hepatic cysts which show low signal intensity in the hepatobiliary phase was not a frequent false positive. Instead, intrahepatic vessels, gallbladder, and heart were common false positives in this study. Among the various false positive detections, extrahepatic structures may be explained by our algorithm in which cropping of images was not performed in our model. Instead, whole MR images were used as input data. However, given that extrahepatic structures such as the heart can be easily discriminated from true HCCs by human readers, this problem may not seriously affect our model’s performance. Unlike our model, most studies dealing with deep learning regarding radiologic imaging require preprocessing of input data. This means that it is necessary to select images containing lesions, which is performed by human readers, and then regions of interest are cropped^8,22,23^. Therefore, the cropped images include only the liver mass and surrounding liver parenchyma or the organ and cropped images are entered into the model as input images. This process may be marginally more complicated and time-consuming. In contrast, our method has advantages over other deep learning models as it does not require the process of selecting an image containing target lesions and cropping of images. In our model, the only thing required is to upload entire hepatobiliary phase MR images. Upon image uploading, our model automatically checks for potential candidate HCC nodules in a very short time. In addition, the location of the HCC as detected by our model was confirmed using a class activation map (CAM)^25^ method. Another advantage of our model is that it works very fast. The average image classification time of 100 images in a single patient was 3.4 seconds, which is faster than a previous study in which 10 seconds was required for the computation of 100 images^23^. However, any direct comparison is limited because the previous study used CT images and the PC performance may be different from ours.

There are several limitations of our study. First, as a preliminary study, only the hepatobiliary phase of gadoxetic acid-enhanced liver MRI was used for the detection of HCCs as the image quality of the arterial phase was frequently affected by transient severe motion artifacts in the arterial phase^26,27^. However, arterial enhancement on cross-sectional imaging is one of the key imaging findings in the diagnosis of HCC. Moreover, given that deep learning with CNN using multiphasic CT images yields higher accuracy than those using single phase CT images^26^, a deep learning model using multiphase MR images may provide higher accuracy. Further study is warranted using other MRI sequences, including arterial phase images. Nevertheless, our preliminary study found that deep learning can be applied in the detection of small HCCs in the hepatobiliary phase of gadoxetic acid-enhanced liver MRI, which showed similar accuracy to that of less-experienced radiologists with a faster interpretation time. Second, our study population for training, validation, and test sets had relatively good liver function as the patient had undergone surgical resection for HCC. In addition, it may be difficult to detect atypical HCCs with our deep learning model as whole tumors included in this study showed low signal intensity in hepatobiliary phase MRI. That implies that our model may work only for patients with good hepatic function and typical HCCs with low signal intensity on hepatobiliary phase MRI. Third, our training data set used MR images obtained from a single vendor MR scanner (Philips Healthcare, Best, The Netherlands), which may have resulted in an overfitting issue and thereby slightly lower accuracy in the validation data set in which a variety of MR scanners from multiple vendors were used. We compared our model with various CNNs networks, and our model seemed to perform well. However, in order to get a clearer conclusion, it is necessary to confirm more cases through additional experiments.

## Methods

### Data collection

The study protocol was in line with ethical guidelines of the 1975 Declaration of Helsinki. This study was approved by the institutional review board (IRB) of Samsung Medical Center (2019-03-101-002), and the IRB waived the requirement to obtain written informed consent from the patients. We reviewed the hepatobiliary phase images of the pre-operative gadoxetic acid-enhanced liver MRI of 549 patients from 2010 to 2014 who were confirmed to have HCC after surgical resection. The equipment used for MR acquisition is listed in Table 5.

**Table 5:** List of data collection equipment.

Manufacturer	Model Name (Tesla)	Number of patients
PHILIPS	Achieva (1.5)	388
PHILIPS	Achieva (3.0)	117
PHILIPS	Ingenia (3.0)	25

### Data categorization

**Figure 8:**
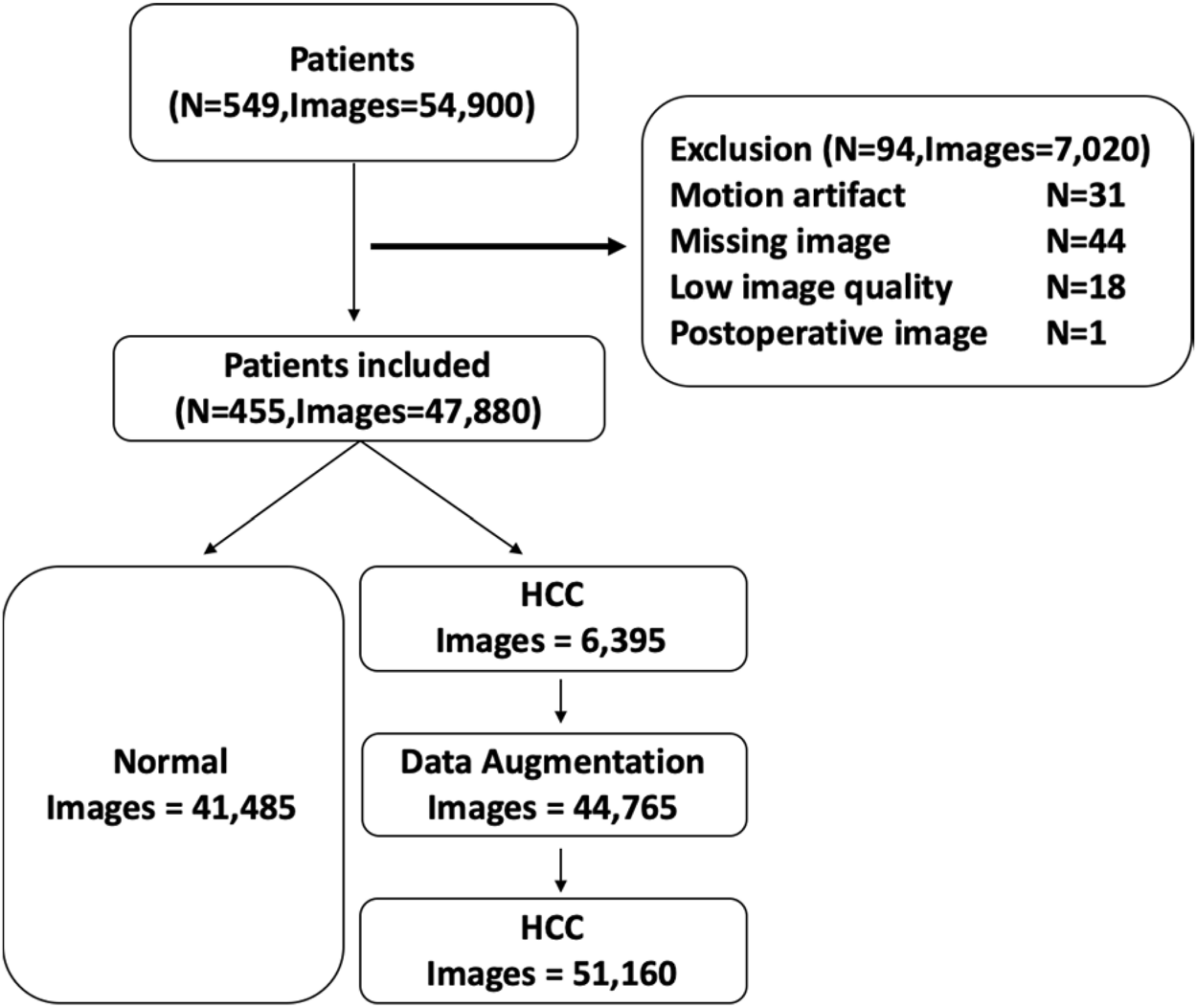
Patient inclusion and exclusion criteria in dataset.

Among 549 patients (442 male patients and 107 female patients with a mean age of 56 years ± SD 9.7), 94 patients were excluded due to severe motion artifacts (n = 31), missing images (n = 44), low image quality (n = 18), or absence of preoperative MR images (n = 1) (Fig. 8). The remaining 455 patients who all had Child-Pugh score A were included in this study. There was a total of 92,645 hepatobiliary phase MR images. They were categorized into no HCC (41,485 images) and HCC (51,160 images) according to whether HCC was present in the image. Among the 92,645 images, 70%, 15%, and 15% were chosen as the training dataset, validation dataset, and test dataset, respectively.

### Data preprocessing and augmentation

Due to the various structures and image sizes included in the MR images, it was difficult to accurately and efficiently learn the characteristics of HCC. Thus, pre-processing that standardizes image size and eliminates unwanted noise was important for improving model learning results and accuracy.

**Figure 9:**
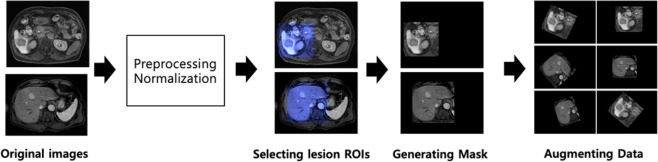
Examples of image augmentation.

Therefore, all images were scaled to 320 × 320 pixels using bicubic interpolation and area interpolation since MR images have diverse pixel sizes (from 256 × 256 to approximately 400 × 400)^28,29^. In addition, among the approximately 100 MR images of each patient, only 3–10 images usually contained an HCC nodule. This relative data shortage problem can lead to excessive over-fitting of the model into classes with large amounts of data in learning. Therefore, we augmented the data in various ways to prevent this. First, the HCC area in the chosen image was extracted using a mask. The mask was generated using a human-annotated label map which distinguished the HCC area. To increase the number of data, the HCC images were augmented using rotation, shift, and zooming as shown in Table 6. We tried not to distort the images since image distortion can reduce performance. Therefore, image rotation was only permitted within 90°. Image shift was performed within 10 pixels for all directions. The image was zoomed from 0.8 to 1.2 times. In addition, shift and zoom were combined. Consequently, we had 44,765 HCC images following the image augmentation process (Fig. 9).

**Table 6:** Data augmentation methods.

Method	Range
Rotate	−90° ≤ rotation angle ≤ 90°
Shift	1–10 pixels
Zoom	0.8–1.2×
Shift and Zoom	Shift (1–10 pixels) and zoom (0.8–1.2×)

### Overall procedure

**Figure 10:**
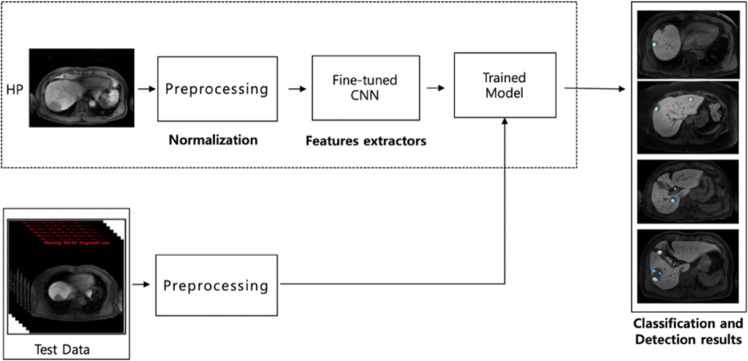
Our proposed Deep Learning system for HCC detection.

The overall process of the proposed deep learning system to detect HCC is explained in Fig. 10. The detailed explanation will be described in the following subsections.

### Convolutional neural network (CNN) architecture

Since there is no solid theory for hyperparameter optimization, we experimented to identify the best combination of the chosen hyperparameters, including batch normalization, dropout, activation function, kernel size, and optimizer. We randomly selected 11,117 images (4,902 no HCC images and 6,215 HCC images) from the training dataset to optimize the CNN architecture. Then we selected 9,449 images (4,167 no HCC images and 5,282 HCC images) as the learning dataset and 1,668 images (736 no HCC images and 932 HCC images) as the validation data set.

### CNN training details

First, all training images were shuffled. Training was terminated when there was no improvement of accuracy in the validation datasets within 20 epochs. The batch size was 128 to balance training quality and convergence speed. The parameters were initialized using the He initializer^17^ and the learning rate was 0.001. ReLU was used as the activation function and the Adam Optimizer was applied. Cross entropy was used for the loss function. A global average pooling layer was applied to the last layer instead of fully-connected layer, since the fully-connected layer loses location information from the image. By using the global average pooling layer, we were able to reduce the size of the parameters and apply the CAM method to generate the heat map. After this layer, softmax was adopted to predict each class. We used a commodity PC (3.7 GHz × 12 Intel Core i7, 64 GB RAM, GeForce GTX 1080Ti 8 G × 2) and TensorFlow V1.8.0.

### Performance evaluation of our model

#### Data collection

**Figure 11:**
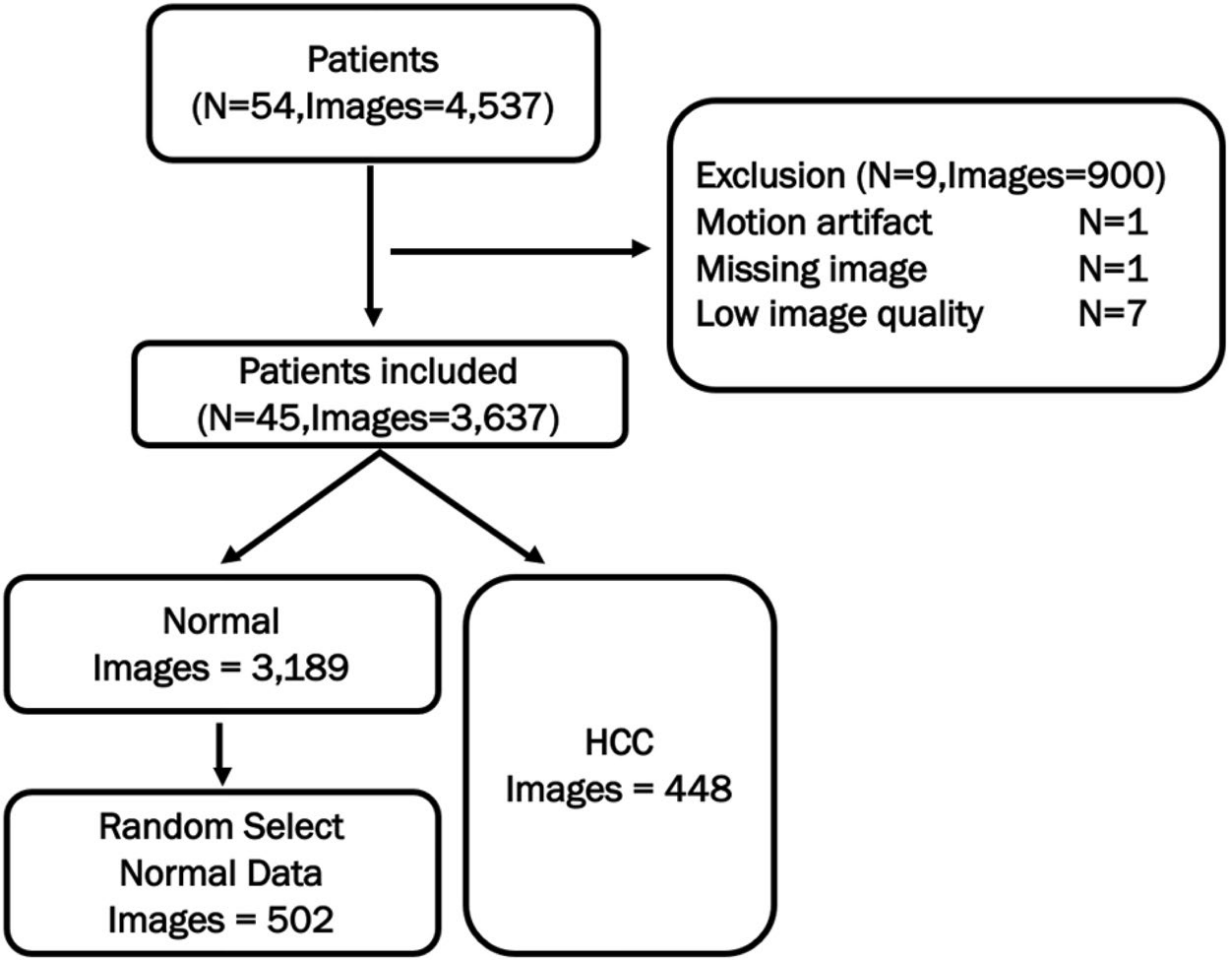
Patient inclusion and exclusion criteria in external validation set.

To verify the performance of our model, we also collected the hepatobiliary phase images from the pre-operation gadoxetic acid-enhanced MRI of 54 patients (42 male and 12 female patients with a mean age of 57 years ± SD 9.6), who had undergone MR imaging at one of four external hospitals from 2015 to 2017. Their histopathologic results were available as they had undergone hepatic surgery in our institution. Among them, nine patients were excluded due to motion artifact (n = 1), missing image (n = 1), or low image quality (n = 7). We randomly selected 502 hepatobiliary phase MR images from 3,189 images with no HCC (Fig. 11). We validated the model using these 502 images and 448 images in which the HCC nodules were included. The equipment used for MR acquisition is listed in Table 7. As in Table 7, the external dataset consisted of MR images obtained with a variety of MR scanners compared to the validation and test datasets.

**Table 7:** List of data collection equipment for external dataset.

Manufacturer	Model Name (Tesla)	Number of patients
PHILIPS	Achieva (1.5)	9
PHILIPS	Ingenia (3.0)	8
PHILIPS	Ingenia CX (3.0)	1
SIEMENS	Avanto (1.5)	6
SIEMENS	Skyra (3.0)	18
SIEMENS	SMS Avanto (1.5)	1
SIEMENS	Verio (3.0)	3
GE	DISCOVERY MR750w (3.0)	2
GE	SIGNA EXCITE (1.5)	2
GE	Signa HDxt (3.0)	4

#### Comparison of performance between our model and human readers

To validate the performance of our model, the sensitivity, specificity, and accuracy of HCC detection were compared between our model and radiologists. Two radiologists (a board-certified abdominal radiologist with 10 years of experience with abdominal imaging and a trainee with 4 years of experience in the department of radiology) participated in this validation study. The two radiologists were blinded to the development of the model and the results of the MR reports and histopathologic results of the external validation datasets. They were only informed that the patients might have risk factors for HCC. Therefore, the radiologists were not aware of the presence, number, or location of the HCCs. They were instructed to record the image number containing HCC nodules in the datasheet when reviewing MR images using a picture archiving and communication system (PACS; Centricity Radiology RA 1000; GE Healthcare, Chicago, IL, USA). They were also requested to record the interpretation time using a stopwatch. The interpretation time was defined as the time between image opening and finishing filling out the datasheet.

#### Image validation

**Figure 12:**
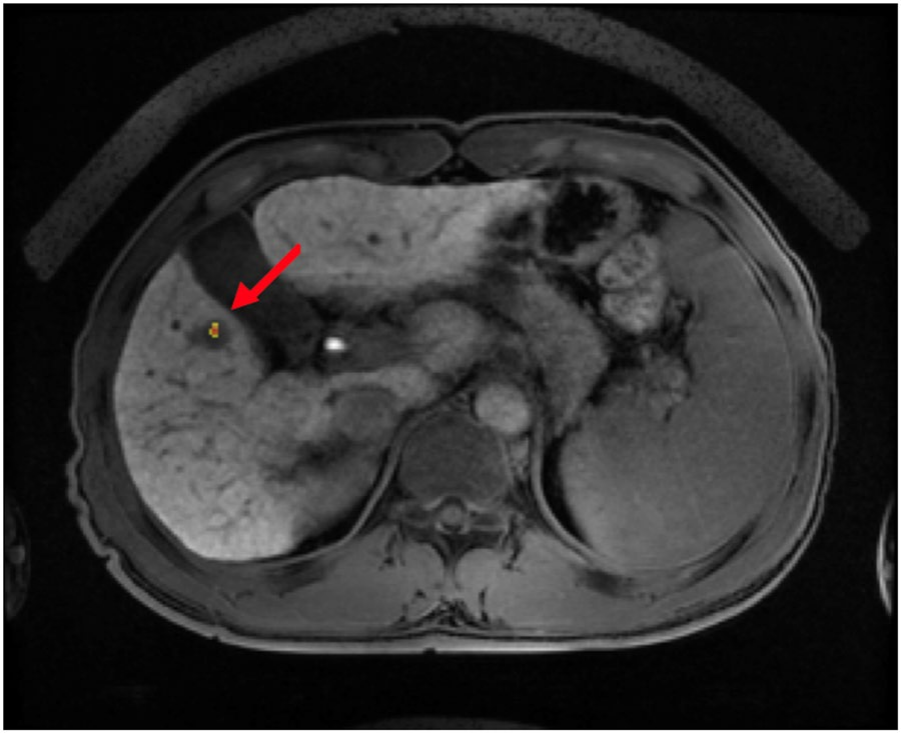
An example of true positive detection (arrow) of HCC by our trained model using CAM method showing the area of interest of the trained model.

To validate the model, we applied a CAM that points to the correct location and provides clues to the physician. Figure 12 shows where the model automatically predicted the HCC.

#### Comparison with other CNNs

We compared our own CNN with three other popular deep learning models including ResNet50^30^, AlexNet^31^, VGG-16^32^ and Inception-ResNetV2^33^. We found that our own CNN architecture outperformed ResNet50, AlexNet, VGG-16 and Inception-ResNetV2. The summary of the result is shown in Table 8 and Supplementary Fig. S1.

**Table 8:** Comparison of CNNs architecture. Our own CNN architecture had the best performance.

Models	NO. of trainable parameters	validation accuracy
ResNet50	23,532,418	93%
AlexNet	43,744,890	57%
VGG-16	241,222,338	56%
Inception-ResNetV2	54,278,690	92%
**Our own CNN**	**1,077,186**	**94%**

## Conclusions

We have created a fully automated, deep learning system that detects and classifies HCCs in gadoxetic acid-enhanced MRI using a new fine-tuned CNN structure. The system classified HCCs six times faster than human readers and achieved 87% sensitivity and 93% specificity in an external validation data set. This result seems to be comparable to the performance of less experienced radiologists. However, our deep learning model has the advantage of detecting very small HCCs better than less experienced radiologists. Finally, for use as a decision support system, we have created a program that categorizes HCCs with a single click and shows the location of candidate HCCs in hepatobiliary phase MR images.

## Supplementary information

Supplementary information.
