# Supplementary material for: Detection of Hepatocellular Carcinoma in Contrast-Enhanced Magnetic Resonance Imaging Using Deep Learning Classifier: A Multi-Center Retrospective Study
Source: Sci Rep. 2020 Jun 11;10:9458. doi: 10.1038/s41598-020-65875-4 (PMC7289813; doi:10.1038/s41598-020-65875-4)

**Detection of Hepatocellular Carcinoma in** **Contrast-Enhanced Magnetic Resonance Imaging Using Deep Learning Classifier: A Multi-Center Retrospective Study**

Junmo Kim1, Ji Hye Min2, Seon Kyoung Kim2, Soo-Yong Shin3,4 & Min Woo Lee1,2

1 *Department of Health and Sciences and Technology, SAIHST, Sungkyunkwan University, 81, Irwon-ro, Gangnam-gu, Seoul, 06351, Korea*

2*Department of Radiology and Center for Imaging Science, Samsung Medical Center, Sungkyunkwan University School of Medicine, 81, Irwon-ro, Gangnam-gu, Seoul, 06351, Korea*

3*Department of Digital Health, SAIHST, Sungkyunkwan University, 115, Irwon-ro, Gangnam-gu, Seoul, 06351, Korea*

4*Big Data Research Center, Samsung Medical Center, 81, Irwon-ro, Gangnam-gu, Seoul, 06351, Korea*

Figure S1: Comparison of CNNs architecture. Our own CNN architecture had the best performance.


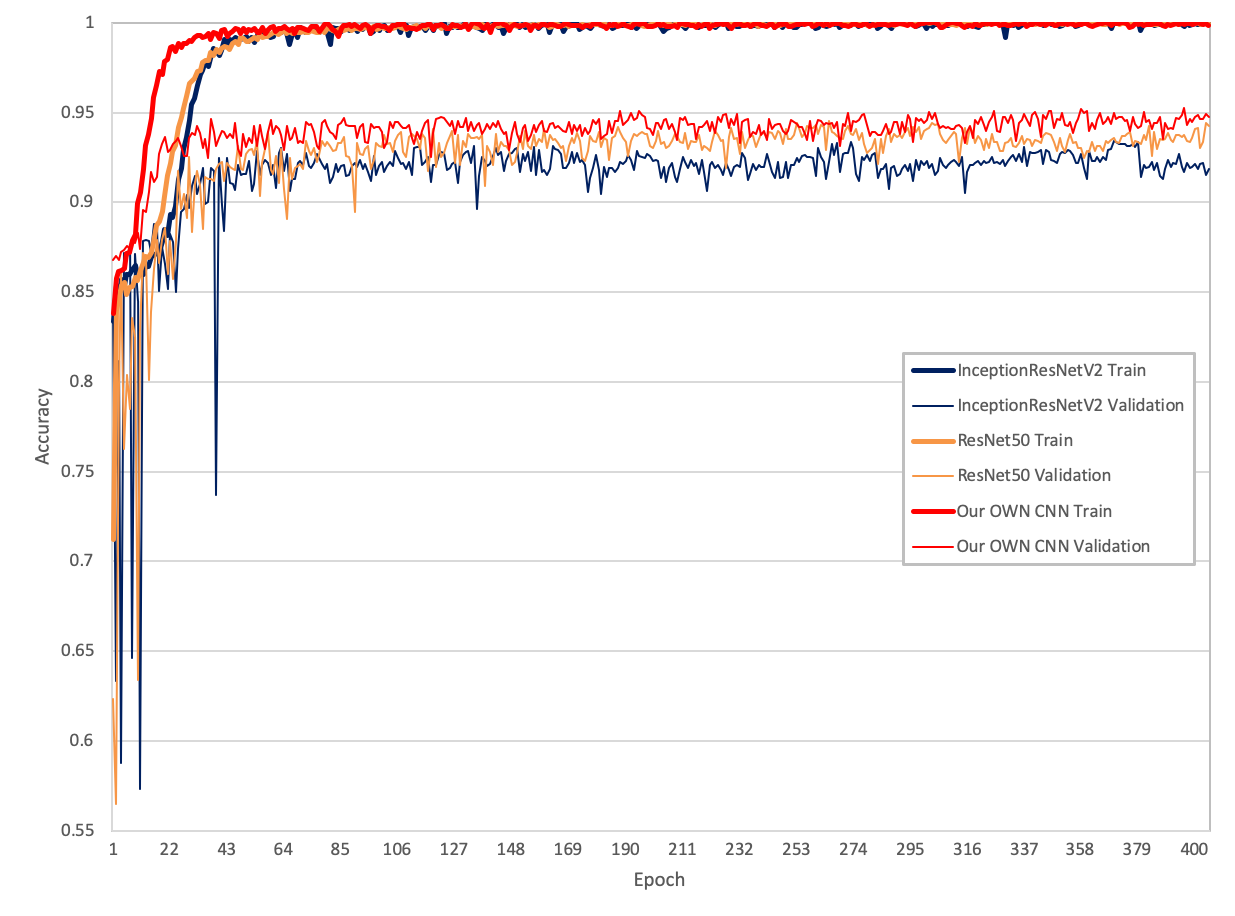

Supplement: Supplementary file 1 — Supplementary information. [file 41598_2020_65875_MOESM1_ESM.docx]
